# SOFA score in gas-forming pyogenic liver abscess: a case series of seven patients with exploratory analysis of severity and mortality

**DOI:** 10.3389/fmed.2026.1886477

**Published:** 2026-08-27

**Authors:** Ning-Bo Xiao, Xiao-dong Zhang, Ze Yu, Jin-Liang Dong

**Affiliations:** 1 Department of General Surgery, Zhoushan Hospital, Wenzhou Medical University, Zhoushan, Zhejiang, China; 2 Department of Intervention, Zhoushan Hospital, Wenzhou Medical University, Zhoushan, Zhejiang, China; 3 Laboratory of Cytobiology and Molecular Biology, Zhoushan Hospital, Wenzhou Medical University, Zhoushan, Zhejiang, China

**Keywords:** diabetes, GFPLA, hypervirulent *Klebsiella pneumoniae*, mortality rate, SOFA score

## Abstract

Gas-forming pyogenic liver abscess (GFPLA) is a rare and highly fatal subtype of bacterial liver abscess, closely related to diabetes and hypervirulent *Klebsiella pneumoniae* infection. This case series analyzed seven patients diagnosed with GFPLA by CT, aiming to explore their clinical characteristics and prognosis. Among the patients, four died, with a total mortality rate of 57.1%, significantly higher than the 27.7%−37.1% reported in the literature. The sequential organ failure assessment (SOFA) scores of the deceased patients at admission were all ≥7, suggesting a potential association between higher SOFA scores and mortality in this small series. The pathogen analysis showed that 85.7% of the cases were hypervirulent *Klebsiella pneumoniae* infections that were positive in the latex agglutination test, and the strains were generally resistant to ampicillin but sensitive to carbapenems and enzyme-inhibitor combination preparations. In terms of treatment, only a few patients received puncture drainage, and some were unable to implement it due to coagulation dysfunction or small abscess volumes. These findings highlight the need for early identification and close monitoring of SOFA scores in patients with GFPLA, particularly those with diabetes, and suggest that a multidisciplinary approach, including effective antibiotics (e.g., carbapenems), active blood glucose control, and timely drainage, may be important for improving outcomes. However, due to the small sample size and exploratory nature of this study, observed associations should be interpreted with caution and require validation in larger cohorts.

## 1 Introduction

Gas-forming pyogenic liver abscess (GFPLA) is a rare and extremely dangerous clinical subtype of bacterial liver abscess, with imaging features characterized by the presence of gas shadows within the abscess cavity (1, 2). Although GFPLA accounts for only 7%−30% of all bacterial liver abscesses (3, 4), its clinical course is often more severe, with rapid onset, swift progression, and a tendency to complicate with infectious shock and multiple organ failure. The reported mortality rate in the literature is as high as 27.7%−37.1% (3, 5).

Epidemiological studies have shown that the occurrence of GFPLA is closely related to host factors, particularly diabetes, which is considered an independent risk factor (6). Hypervirulent *Klebsiella pneumoniae* (hvKP) is the primary pathogen of this disease, and its positive string test and carriage of virulence genes such as rmpA are closely associated with invasive infection syndrome (7, 8). However, due to the sporadic nature of the disease throughout the year, clinicians still lack sufficient experience in its early identification, disease severity assessment, standardized diagnosis and treatment strategies, leading to treatment delays and poor prognosis. Existing studies have mostly focused on comparing the clinical characteristics of GFPLA with non-gas-forming liver abscesses (4), but clinical analyses specifically targeting the GFPLA population, particularly quantifying association between disease severity and prognosis, remain limited.

The study reported clinical data of seven patients diagnosed with GFPLA by CT, of which four died, with a case fatality rate as high as 57.1%, significantly higher than the average level reported in previous literature. This case series aims to improve clinicians' vigilance toward this disease by providing a detailed analysis of the clinical characteristics, etiology, drug sensitivity results, and treatment outcomes of these patients, with a focus on exploring the value of the SOFA score in assessing disease severity and predicting mortality, thereby offering a scientific basis for early recognition, timely intervention, and reduction of the mortality rate.

## 2 Case presentation

### 2.1 Patient information

This study is a retrospective analysis aimed at investigating the clinical characteristics and prognostic risk factors of gas-forming pyogenic liver abscess. The study subjects were seven patients diagnosed with gas-forming pyogenic liver abscess who were admitted to Zhoushan Hospital between January 2019 and April 2021. All cases were confirmed by computed tomography (CT) imaging examination. Inclusion criteria included complete clinical and epidemiological data, while exclusion criteria were non-bacterial infectious liver abscess, secondary liver abscess, and incomplete clinical data.

### 2.2 SOFA score calculation

The sequential organ failure assessment (SOFA) score was calculated for each patient at the time of admission and reassessed daily during hospitalization. The score comprises six organ systems: respiratory (PaO_2_/FiO_2_ ratio), coagulation (platelet count), the hepatic (total bilirubin), cardiovascular (mean arterial pressure or requirement for vasopressors), renal (serum creatinine or urine output), and neurological (Glasgow Coma Scale). Each domain is scored from 0 to 4, yielding a total score between 0 and 24. For the purpose of this study, the SOFA score obtained within the first 24 h of admission (the initial SOFA score) was used for severity stratification and prognostic analysis. Dynamic changes in SOFA score during hospitalization were also monitored.

### 2.3 Clinical manifestation

Among the seven patients included in the study, six were male and one was female. The average age was (58 ± 13.3) years. The age distribution was mainly among young and middle-aged individuals (six cases aged 37–69 years), and there was one patient aged 82 years old. The occupations included farmers, fishermen, workers, housekeepers, and unemployed individuals (Table 1). Six patients had underlying diseases, among which four had diabetes and two had chronic liver diseases. In terms of clinical manifestations, the most common symptom was fever (six cases), followed by right upper abdominal pain (five cases), chills (four cases), and shock (four cases). Routine laboratory tests and basic disease screenings are shown in Table 1, and the patient's treatment information is displayed in the Supplementary Table 1. According to the sequential organ failure assessment (SOFA) score, five patients met the criteria for severe infection (organ dysfunction), and among them, four cases had a SOFA score of ≥7.

**Table 1:** Patient information.

Patient	1	2	3	4	5	6	7
Age	62	82	37	69	43	52	61
Gender	Man	Woman	Man	Man	Man	Man	Man
Occupation	Farmer	Unemployed	Fisherman	Farmer	Worker	Fisherman	Unemployed
Abdominal pain	+	+	–	+	+	–	+
Fever	+	+	–	+	+	+	–
Shivering	+	–	–	+	+	+	–
Shock	+	–	–	+	+	+	–
White blood cell count is elevated	+	+	–	–	–	+	–
Elevated neutrophils	+	+	–	+	+	+	+
Thrombocytopenia	+	–	–	+	+	+	–
Hypoproteinemia	+	+	–	+	+	+	+
Increased γ-GT	+	+	–	–	+	–	+
Increased serum creatinine	–	–	–	+	+	–	+
LDHA elevated.	+	–	–	+	+	+	–
Blood culture (*Klebsiella pneumoniae*)	+	–	–	+	+	–	+
Culture of drainage fluid (*Klebsiella pneumoniae*)	+	+	–	–	–	+	–
Location of the abscess	Left	Right	Left	Right	Right	Left	Right
Diabetes	–	–	–	+	+	+	+
Chronic liver disease	+	–	–	–	+	–	–
Puncture and drainage	+	+	–	–	–	+	–
SOFA score	7	4	1	11	11	7	1
Result	Death	Cure	Cure	Death	Death	Death	Cure

### 2.4 Diagnostic assessment

**Figure 1:**
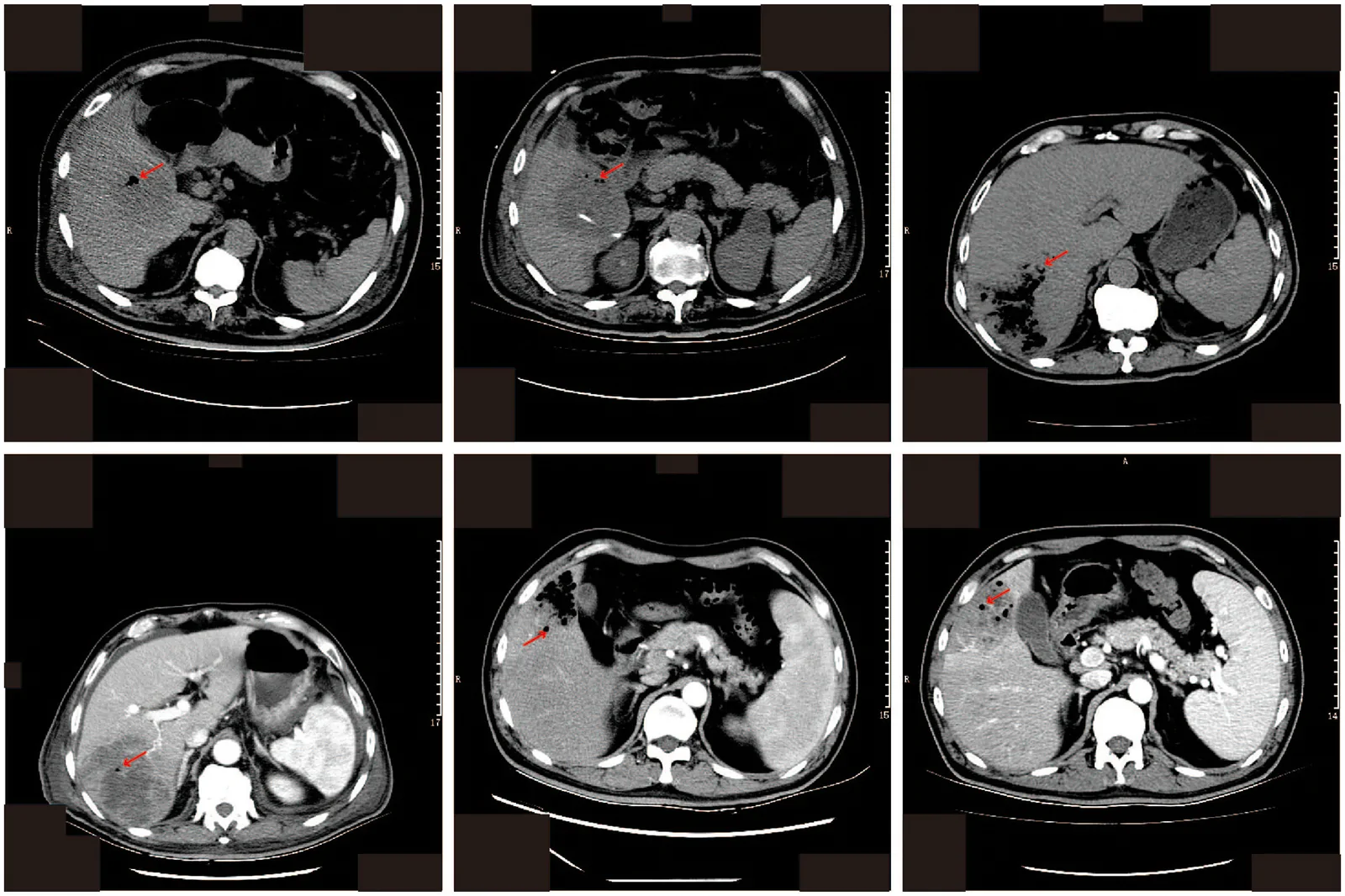
CT manifestations of patients with gas-producing liver abscesses. The red arrow points to the gas.

All patients were diagnosed with liver abscess accompanied by intrahepatic gas accumulation via CT examination (Figure 1). Etiological examination results showed that six patients were confirmed to have *Klebsiella pneumonia*e infection, of which four were confirmed by blood culture, and three were confirmed by abscess drainage fluid culture (one patient had consistent results from both blood culture and drainage fluid culture). All isolated strains tested positive for the string test. Species identification was performed using the VITEK 2 system (bioMérieux, Marcy-l'Étoile, France). Antimicrobial susceptibility testing (AST) was conducted using the VITEK 2 AST-GN card (bioMérieux) method, according to the manufacturer's instructions. Results were interpreted based on Clinical and Laboratory Standards Institute (CLSI) guidelines. *Klebsiella pneumoniae* ATCC 700603 and *Escherichia coli* ATCC 25922 were employed as quality control strains for AST. The string test was performed using standard methodology, and a positive result was defined as the formation of a viscous string >5 mm when a bacteriological loop was dragged upward from a colony grown on blood agar.

Antimicrobial susceptibility testing indicated that the isolated strains were sensitive to imipenem, Tigecycline, piperacillin-tazobactam, amoxicillin-clavulanic acid, Cefoperazone and sulbactam, Ceftazidime, cefepime, Gentamicin, and amikacin. However, the six strains were resistant to ampicillin, and some strains showed resistance to cefazolin, Ceftriaxone, piperacillin, ampicillin-sulbactam, and chloramphenicol.

### 2.5 Treatment measures

All patients received empirical antibiotic treatment upon admission, based on the results of the antimicrobial susceptibility test (blood culture), the antibiotic treatment regimens have been adjusted, including piperacillin-tazobactam, ceftazidime-sulbactam, meropenem and meropenem, etc., for a duration ranging from 4 to 14 days. For the liver abscess itself, three patients underwent ultrasound-guided percutaneous liver abscess drainage. Reasons for not performing drainage included small abscess size (two cases) and significantly reduced platelet count with a bleeding risk (two cases). Throughout the treatment process, emphasis was placed on the management of primary diseases, particularly active blood glucose control in patients with comorbid Diabetes Mellitus.

### 2.6 Follow-up and Outcomes

In this study cohort, the overall prognosis was poor, with a total of four deaths among seven patients. The deaths were all concentrated in the group of patients with higher SOFA scores (≥7 points). Combining the treatment process and outcome analysis, early identification of septic shock, timely etiological diagnosis and targeted anti-infection therapy guided by drug sensitivity testing, as well as timely implementation of abscess drainage, are key factors affecting prognosis. In addition, active control of blood glucose and early transfer to the intensive care unit (ICU) for organ function support therapy are of great significance for improving the prognosis of such critically ill patients.

## 3 Discussion

Gas-forming pyogenic liver abscess (GFPLA), as a rare but extremely dangerous subtype of bacterial liver abscess, has been confirmed to have a high mortality rate in multiple studies. Literature reports indicate that the mortality rate of GFPLA is as high as 27.7%−37.1% (3), while in this case series, four out of seven patients died, resulting in a mortality rate of 57.1%, which is significantly higher than previously reported levels. This difference may be partly attributed to the extremely critical condition of the patients in this group upon admission. All four patients with a SOFA score ≥7 died, which is highly consistent with recent studies emphasizing that the SOFA score is an independent risk factor for invasive syndrome and mortality in patients with liver abscess (9). In terms of etiology, six cases (85.7%) in this group were confirmed to be infected with hypervirulent *Klebsiella pneumoniae*, and all strains tested positive for the string test. This aligns with the global trend where hypervirulent *Klebsiella pneumoniae* (hvKP) has become the main pathogen of GFPLA (10). Notably, the strains in this group were generally resistant to ampicillin, with some showing resistance to cefazolin and ceftriaxone, while carbapenems and enzyme inhibitor combination preparations remained highly sensitive. This is consistent with the antimicrobial susceptibility profile of hvKP, which is typically sensitive to most commonly used antibiotics except ampicillin (11–13).

At the therapeutic level, only three cases (42.9%) in this group underwent percutaneous drainage of liver abscess, while two cases did not undergo drainage due to small abscess size and two cases due to thrombocytopenia. However, a recent metabonomics study on gas-forming liver abscess indicated that Diabetes Mellitus is an independent risk factor for GFPLA, and gas formation is more likely associated with metabolic changes in the liver microenvironment induced by Hyperglycemia, rather than being solely determined by flora structure (6). This suggests that for GFPLA patients with concurrent Diabetes Mellitus, active glycemic control may have greater pathophysiological significance than reliance on drainage alone. Furthermore, the two cases in this group where drainage was abandoned due to thrombocytopenia highlight the clinical dilemma of balancing bleeding risk and drainage benefits in critically ill patients. Although studies have shown percutaneous drainage is associated with reduced short-term mortality, the feasibility of the surgical technique does not equate to survival benefits, especially in patients who have already developed multiple organ dysfunction. Therefore, for GFPLA patients with rapidly increasing SOFA scores, prompt initiation of potent anti-infective therapy (e.g., carbapenems) and active transfer to the ICU for organ support should be considered, while closely monitoring Blood coagulation function to identify the optimal timing for drainage.

The key clinical management insights revealed by this case series first lie in a profound understanding of diagnostic pitfalls. Early clinical manifestations of GFPLA are often nonspecific, presenting only as fever and unexplained abdominal pain, which can easily be misdiagnosed as common infections or acute abdomen, thereby delaying treatment (14). In this series, all deceased patients had a SOFA score ≥7, while all surviving patients had a score ≤ 4. This stark contrast strongly suggests that dynamic monitoring of the SOFA score is a crucial early warning tool for identifying disease deterioration. Clinicians should be vigilant: for middle-aged and elderly patients with diabetes, even if initial imaging (such as ultrasound) is negative, once clinical deterioration or lack of improvement in inflammatory markers occurs, a decisive repeat examination using bedside ultrasound (POCUS) or CT should be performed to avoid missed diagnosis. Furthermore, the close association between gas formation and hyperglycemia in this case series echoes the hypothesis proposed by metabonomics studies, that hyperglycemia- induced changes in the hepatic microenvironment (such as tissue hypoxia and enhanced glycolysis) promote the metabolic activity of gas-producing bacteria. This is not only a pathophysiological mechanism of GFPLA but also constitutes a unique diagnostic clue (15).

Furthermore, this case series highlights several easily overlooked teaching points in comprehensive management strategies. At the level of anti-infective treatment, the widespread drug resistance of this group of strains to ampicillin and some cephalosporins warns that empirical antibiotic selection must abandon these drugs. Notably, “hepatic gas” on imaging has been confirmed as an independent risk factor for multidrug-resistant bacterial infection (aOR: 26.0) (16), meaning that for GFPLA patients, initial anti-infective regimens should cover drug-resistant bacteria, and even carbapenems may be directly considered. In terms of source control, although puncture drainage is the standard treatment, cases in this group where drainage was abandoned due to low platelet count reveal the difficult trade-off between coagulation disorders and infection control. For such patients, drainage should not be mechanically insisted upon; instead, priority should be given to correcting coagulation disorders through measures such as platelet transfusion, while simultaneously intensifying antibiotic therapy and systemic support. Finally, all deceased patients in this group did not receive effective blood glucose control, and the literature clearly indicates that controlling blood glucose is a core element in improving prognosis. Therefore, the management of GFPLA is by no means a simple surgical or anti-infective issue, but a complex systemic project requiring dynamic assessment and multidisciplinary collaboration (including the Endocrinology Department and ICU), with its core lying in early identification of critical tendencies, precise selection of antibiotics, and active management of host factors (especially blood glucose and coagulation function). The data presented in our study support the selection of antibiotics for patients with liver abscesses.

This case series, through a retrospective analysis of clinical data from seven patients with GFPLA, further confirms the high lethality of this disease and its close association with host factors such as SOFA score, hypervirulent *Klebsiella pneumoniae* infection, and diabetes mellitus. However, this study has significant limitations. As a single-center, small-sample retrospective case series with only seven cases, key data such as mortality rate (57.1%) may be subject to considerable bias, failing to adequately represent the overall prognostic spectrum of GFPLA. Besides, due to the extremely small sample size and the absence of a control group, statistical analysis cannot be conducted. Second, the retrospective design inherently makes it difficult to avoid information bias; for example, details of blood glucose control, timing of antibiotic prophylaxis, and dose adjustments in some patients were not fully standardized in recording, limiting in-depth exploration of the causal relationship between treatment strategies and prognosis. Finally, due to the lack of a concurrent control group with non-gas-forming liver abscesses, this study cannot directly quantify the independent impact of gas formation on prognosis.

Despite the aforementioned limitations, this series provides important reflections for clinical practice. The success of GFPLA treatment often depends on the time window from the first medical contact to the initiation of comprehensive intervention. The common feature of the deceased cases in this group is that they were already in a state of high SOFA scores upon admission, suggesting that the lack of an early warning system is the core link leading to adverse outcomes. Therefore, clinicians should abandon the “rare disease” mindset inertia regarding GFPLA. For middle-aged and elderly patients with diabetes, unexplained fever and abdominal pain, and sharply elevated inflammatory markers, abdominal CT should be proactively used as a first-line screening tool, and SOFA scores should be dynamically monitored to identify early signs of organ dysfunction. In the future, it is necessary to conduct multi-center, prospective cohort studies with sufficient sample sizes and control groups to further clarify the independent prognostic risk factors for GFPLA and to verify whether early intensive intervention strategies driven by SOFA scores can effectively reduce mortality.

## 4 Conclusion

In summary, patients with gas-forming pyogenic liver abscess present with severe conditions, rapid progression, and high mortality rates, and are highly prone to septic shock leading to multiple organ failure or even death, requiring high clinical vigilance. In this small case series, all deceased patients had SOFA scores ≥7 at admission, suggesting a potential association between higher SOFA scores and mortality in this exploratory analysis. However, due to the limited sample size, this observation cannot be interpreted as establishing the SOFA score as a predictor of mortality, and further validation in larger cohorts is needed. For those with elevated blood glucose, active glycemic control is essential, and timely liver abscess puncture drainage, along with early and appropriate use of antibiotics to control infection, as well as systemic supportive therapy, are particularly critical steps in the treatment process.

## Data Availability

The original contributions presented in the study are included in the article/Supplementary material, further inquiries can be directed to the corresponding authors.
